# Immunosuppressive cells in acute myeloid leukemia: mechanisms and therapeutic target

**DOI:** 10.3389/fimmu.2025.1627161

**Published:** 2025-07-23

**Authors:** Mengnan Liu, Mengting Yang, Yue Qi, Yuting Ma, Qulian Guo, Ling Guo, Chunyan Liu, Wenjun Liu, Lan Xiao, You Yang

**Affiliations:** ^1^ Department of Cardiovascular Medicine, Affiliated Traditional Chinese Medicine Hospital, Southwest Medical University, Luzhou, China; ^2^ Department of Pediatrics (Children Hematological Oncology), Birth Defects and Childhood Hematological Oncology Laboratory, The Affiliated Hospital of Southwest Medical University, Sichuan Clinical Research Center for Birth Defects, Luzhou, Sichuan, China; ^3^ Department of Pediatrics, Southwest Medical University, Luzhou, Sichuan, China

**Keywords:** acute myeloid leukemia, regulatory T cells, regulatory B cells, myeloid-derived suppressor cells, leukemia-associated macrophages, leukemia-associated neutrophils

## Abstract

Immunotherapy has emerged as a cornerstone strategy for augmenting therapeutic efficacy in acute myeloid leukemia (AML). The immunosuppressive AML microenvironment, characterized by profound immune dysfunction, critically impairs anti-leukemic immune surveillance. This immunologically hostile niche is principally governed by specialized immunosuppressive cell populations—notably regulatory T cells (Tregs), myeloid-derived suppressor cells (MDSCs), leukemia-associated macrophages (LAMs), and regulatory B cells (Bregs)—which collectively establish an immune-privileged sanctuary for leukemic cells. This review critically examines three fundamental aspects of these immunosuppressive regulators in AML pathogenesis: (1) their recruitment dynamics within the leukemic niche, (2) the molecular mechanisms underlying their immunosuppressive functions, and (3) current and emerging therapeutic approaches designed to neutralize their inhibitory effects. Through this comprehensive analysis, we aim to provide a mechanistic framework for developing more effective immunotherapeutic interventions against AML.

## 1 Introduction

Acute myeloid leukemia (AML) is a highly aggressive hematologic malignancy characterized by uncontrolled clonal proliferation of immature myeloid cells, resulting in the accumulation of abnormal blast cells in the bone marrow (BM) and impairment of normal hematopoietic function (1). AML is the most prevalent form of leukemia in adults, with an annual incidence rate of approximately 3 to 5 cases per 100,000 individuals (2–4). AML patients typically have a poor prognosis, marked by a short survival time and unsatisfactory clinical outcomes. AML is a profoundly heterogeneous hematologic malignancy with multifaceted pathophysiology involving: genomic instability and mutational accumulation, oncogenic fusion events, epigenetic reprogramming, immune dysregulation and inflammatory cascades, apoptosis resistance mechanisms, metabolic pathway derangements, cellular senescence evasion, growth suppression circumvention, and sustained proliferative signaling (5–12).

Current AML treatment strategies include conventional chemotherapy, targeted therapies (FLT3/IDH/BCL-2 inhibitors), hematopoietic stem cell transplantation, and emerging immunotherapies (CAR-T, checkpoint inhibitors) with microenvironment-modulating approaches (13). Although advancements in treatment have led to improvements in AML prognosis, challenges such as chemoresistance, relapse, and refractory disease persist as significant barriers (14).

Emerging evidence underscores the pivotal role of bone marrow niche dysregulation in AML pathogenesis (9, 10, 15). During disease progression, the microenvironment undergoes profound cellular and functional remodeling, creating a permissive ecosystem that sustains leukemic cell survival (16). Notably, the AML microenvironment exhibits prominent immunosuppressive characteristics (17). Key immunosuppressive cell populations—including regulatory T cells (Tregs), myeloid-derived suppressor cells (MDSCs), leukemia-associated macrophages (LAMs), regulatory B cells (Bregs) and leukemia-associated neutrophils (LANs)—employ diverse mechanisms to facilitate immune evasion by leukemic cells. Therapeutic targeting of these immunosuppressive populations represents a promising strategic approach for AML immunotherapy. A comprehensive understanding of the regulatory networks of these immunosuppressive cells is crucial for developing novel immunotherapeutic strategies. This review provides a comprehensive analysis of the role and mechanisms of crucial immunosuppressive cells within the AML microenvironment, including Tregs, MDSCs, LAMs, Bregs and LANs, to serve as a reference for future research in this field.

## 2 The famous immunosuppressive cell: regulatory T cell

### 2.1 The phenotype of Treg

Tregs represent a heterogeneous population of T cells, exhibiting diverse origins, phenotypes, and effects. The traditional classification of Tregs comprises two primary subsets: thymic Tregs (tTregs), also referred to as natural Tregs (nTregs), and peripheral Tregs (pTregs), alternatively known as induced Tregs (iTregs) or adaptive Tregs (aTregs), depending on their distinct sources (18). In the thymus, a subset of CD4 single-positive autoreactive cells successfully undergo negative selection by expressing FOXP3, leading to their differentiation into thymic Tregs (tTregs). These tTregs make up approximately 5% to 10% of CD4+ T cells present in the peripheral blood (PB) (19, 20). pTregs are generated from naive CD4+ T cells in the peripheral tissues in response to various stimuli, including antigens, as well as factors like TGF-β and IL-2 (21, 22). Interestingly, Treg cells display a relatively anergic state and are unable to produce IL-2 due to the transcriptional repressive effects of FOXP3 (23), despite the fact that IL-2 is essential for the generation, survival, and activation of Tregs (24). Aside from the conventional CD4+ Treg cells mentioned previously, several other T cell subsets have been identified to possess immunosuppressive capabilities. These include CD8+ T cells (25), IL-17+ Treg cells (26), ICOS+ Treg cells (27), Type II NKT cells (28, 29), and γδT cells (30). A comprehensive summary detailing the phenotypes of T cells exhibiting regulatory properties can be found in **Table 1**.

**Table 1:** Phenotypes of T cells with regulatory properties.

Cell type			Phenotype	Reference
CD4+	nTreg		CD4+CD25+FOXP3+CTLA-4+CD45RO+CD127^low^	(225)
	iTreg	Th3	CD4+CD25±FOXP3±CD45RO+CTLA-4+	(226)
		Tr1	CD4+CD25±FOXP3±CD45RO+CTLA-4−	
		TGF-β/IL-10 double-positive Treg	CD4+CD25−FOXP3−	
		IL-17+ Treg	CD4+CCR9+CD25+CD127^dim/−^	(227)
CD8+			CD8+FOXP3+	(228)
			CD8+CD103+	
			CD8+CD28−	
			CD8+CD122+CD49d+	
			CD8+CD122^high^Ly49+	
γδT cell			FOXP3+TCRγδ+	(51, 52)
CD4-CD8- double negative Treg			TCRαβ+/γδ+CD3+CD4−CD8−NK1.1−	(229, 230)
Type II NKT cell			CD3+CD56+CD161+TCRγδ−TCRVα7.2−TCRVα24−	(29, 231)

Currently, the primary markers employed for the identification of conventional Tregs are CD25^high^, CD127^low/−^, and FOXP3+ (31). Furthermore, several supplementary molecules, including CD45RA (32), CD39/CD73 (33), CD26 (34), CD6 (35), NRP-1 (36), TIM-3 (37), and others (38), can serve as surface markers for Tregs.

### 2.2 Treg accumulation and its mechanisms in AML

Numerous studies have demonstrated an elevated frequency of Tregs in the BM and PB of AML patients. The heightened accumulation of Tregs within the AML microenvironment not only facilitates the development and advancement of AML but also amplifies treatment resistance and the likelihood of relapse.

#### 2.2.1 Elevated Tregs observed in AML occurrence, drug resistance, and relapse

Elevated percentages of Tregs contribute to the establishment of an immunosuppressive microenvironment in AML, providing favorable conditions for the survival and proliferation of malignant AML cells. Consequently, this immunosuppressive milieu plays a facilitating role in the progression and pathogenesis of the disease. Wang et al. discovered that individuals newly diagnosed with AML exhibited an increased proportion of CD4+CD25^high^ Tregs in both PB and BM. Notably, these Tregs displayed a more robust state of renewal, characterized by heightened rates of proliferation and apoptosis, when compared to healthy donors (39). The elevated presence of Tregs in newly diagnosed AML patients results in a reduced ratio of Th17/Treg cells. This finding confirms the immunosuppressive polarization of the bone marrow microenvironment in AML (40). In the PB of AML patients, circulating T follicular regulatory cells (cTfr), defined as CD4+CXCR5+PD-1+FOXP3+, were elevated, indicating increased suppression of B cell responses (41). Additional studies have consistently identified greater proportions of Tregs in the BM and PB of patients diagnosed with AML compared to healthy control subjects (42, 43). These findings underscore the abundant presence of Tregs in AML and their role in establishing an immunosuppressive microenvironment. Contrary to previous beliefs, a recent report suggests that the proportion of Tregs in the BM is similar between individuals with AML and healthy donors. However, it was observed that AML patients exhibit higher proportions of effector Tregs (CD45RA− Tregs). Furthermore, the study found a significant increase in PD1+/TIGIT+ Tregs in the BM of AML patients with a high leukemia burden (44). This suggests that the AML microenvironment may intensify the regulatory function of Tregs, and the number of Tregs present is influenced by the extent of leukemia burden.

In addition to the involvement in pathogenesis, Tregs have also been demonstrated a connection to chemotherapy resistance and disease relapse. Szczepanski et al. conducted a study that reaffirmed the observation of elevated percentages of Tregs and their suppressive activity in the PB of AML patients. Remarkably, the study found that patients with a lower frequency of Tregs at the time of diagnosis exhibited a more positive response to induction chemotherapy (45). Ersvaer et al. observed persistent high frequency of Tregs in AML patients both prior to chemotherapy and throughout the period of cytopenia induced by intensive chemotherapy. Additionally, these proportions remained elevated during the regeneration phase following treatment (46). Moreover, several other research groups have reported an increase in Treg expansion in the PB during the recovery of lymphocytes after intensive chemotherapy and during cytotoxic maintenance chemotherapy (47, 48). Several studies have indicated that patients with AML who achieved complete remission (CR) experienced a notable decrease in Treg frequency compared to those at the time of diagnosis (42, 49), and Zhang et al. further observed a sudden increase in Tregs during relapse, suggesting that monitoring Treg frequency after achieving CR could serve as a valuable predictor of relapse (49). Additionally, findings from a phase IV clinical trial (NCT01347996) revealed that the accumulation of Tregs in the PB as a result of immunotherapy with HDC/IL-2 is associated with the risk of relapse in AML. In cycle 3 of the treatment, a decrease in Treg accumulation was indicative of a lower risk of relapse, supporting the notion that the prolonged presence of Tregs may adversely affect the prognosis of AML (50). Strikingly, in Szczepanski’s study, patients who achieved CR still maintained an increased frequency of Tregs, which was counterintuitive and inconsistent with the observations of other researchers. They proposed an interesting conclusion that Tregs are resistant to conventional chemotherapy (45). In addition to the conventional Tregs, studies have also shown that γδ Treg cells are increased in AML patients and correlated with unfavorable clinical outcomes (51, 52). Therefore, the assessment of Treg frequency holds considerable importance in understanding the progression of leukemia, treatment response, and prognosis in AML patients. A compilation of studies focusing on Treg accumulation in AML can be found in **Supplementary Table S1**.

#### 2.2.2 Accumulation mechanisms of Tregs in AML microenvironment

**Figure 1:**
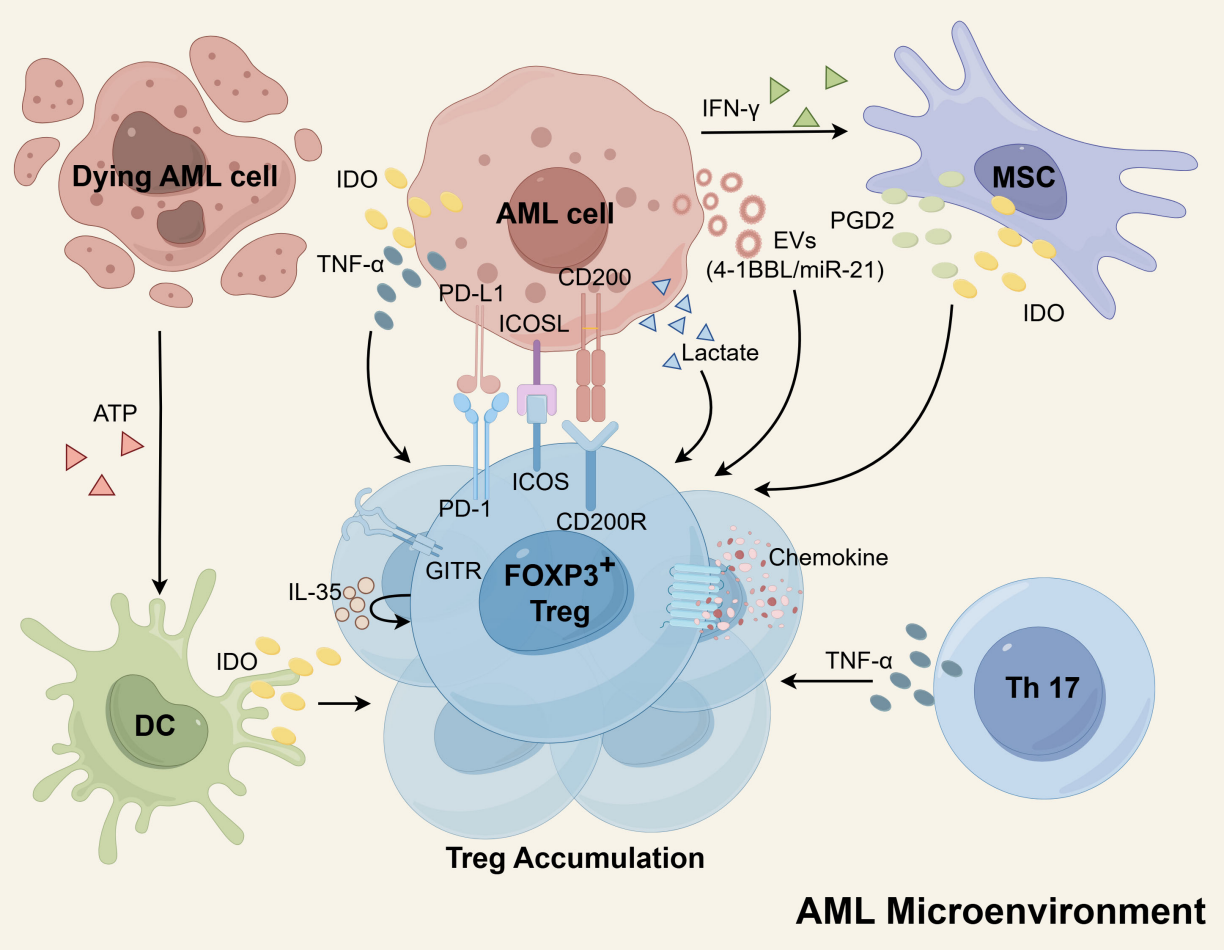
The mechanisms of Treg cells accumulation in the AML microenvironment. The secretion of EVs by AML cells plays a role in increasing Tregs, as these EVs contain molecules such as miR-21 and 4-1BBL that promote Treg expansion. Additionally, AML cells, DCs, and MSCs can produce IDO, which induces proliferation of Tregs. MSCs also release PGD2 to enhance Treg numbers. Both Th17 cells and AML cells express TNF-α, which supports the expansion of Tregs. Furthermore, Tregs themselves express high levels of IL-35, which can further amplify Treg proliferation. The interaction between AML cells and Tregs through receptor-ligand interactions, including PD-L1/PD-1, ICOSL/ICOS, and CD200/CD200R, also promotes Treg expansion. Tregs possess enhanced chemokine receptors, facilitating robust migration and contributing to their aggregation. Moreover, Tregs have a metabolic advantage as they can utilize lactate for metabolism, indirectly contributing to their accumulation. Schematic figure was drawn by Figdraw (www.figdraw.com).

Numerous studies have elucidated the mechanisms underlying the accumulation of Tregs within the microenvironment of AML. These well-established mechanisms encompass the secretion of specific factors, interactions between receptors and ligands, chemotactic effects, and metabolic advantages (**Figure 1**). Subsequently, we will delve into each of these mechanisms in detail.

Recent findings have revealed that extracellular vesicles (EVs) derived from AML cells and containing 4-1BBL play a pivotal role in augmenting the expression of FOXP3 and the effector phenotype in Tregs, thereby bolstering their activity. Treg cells actively internalize EVs carrying the costimulatory ligand 4-1BBL, resulting in the upregulation of STAT5 and the suppression of mTOR-S6 signaling. Consequently, this process promotes the immunosuppressive effector Treg cells (53). In addition, miR-21 originating from AML-derived EVs has been demonstrated to promote the expression of genes recognized as markers for Tregs and immunosuppression. These genes include *IL-10*, *FOXP3*, *CTLA-4*, and others. Intriguingly, the transfer of miR-21 into leukemia-infiltrating T lymphocyte cells yielded the acquisition of a Treg cell phenotype, accompanied by a notable increase in FOXP3 levels in AML (54).

Indoleamine 2,3-dioxygenase (IDO) is an enzyme with immunomodulatory properties that facilitates the conversion of tryptophan (Trp) into kynurenines (Kyn). These Kyn metabolites have the ability to promote the generation of Treg (55). The generation of this inducible Treg can be significantly hindered by the IDO inhibitor, 1-methyl tryptophan (1-MT) (56, 57). Arandi et al. revealed that elevated expression of IDO in patients with AML may contribute to an increase in the number of Treg (58). Furthermore, *in vitro* studies have demonstrated the presence of functionally active IDO proteins within AML cells, which have the capability to stimulate the proliferation of Treg (56, 59). In a study by Curti et al., it was reported that a notable proportion of primary blast cells derived from adult patients with AML constitutively express the active form of IDO protein (60). Conversely, a multicenter study involving pediatric AML patients indicated that blast cells do not exhibit constitutive expression of IDO protein. However, functional IDO protein was found to be upregulated in approximately half of the AML samples in response to IFN-γ stimulation (61). IDO is an IFN-γ-inducible enzyme, whose expression is transcriptionally activated through the JAK-STAT1 signaling pathway in coordination with the transcription factor IRF1 (62). These studies suggest that regardless of whether IDO protein is constitutively expressed or induced, it is evident that AML cells have the capability to produce and release IDO protein. This leads to an elevation of IDO concentration within the microenvironment, consequently promoting the expansion of Treg. Additionally, dendritic cells (DCs) are known to express functional IDO protein, which can hinder the T-cell response by facilitating the expansion of Tregs (62). DCs derived from AML cells have been suggested as potential leukemia vaccines due to their increased immunogenicity. However, one challenge is that these DCs show upregulation of IDO, which can negatively impact immune responses by activating powerful Tregs (63). Clinical sample analysis has demonstrated that adenosine triphosphate (ATP) released by dying AML cells, specifically those targeted by chemotherapy, plays a role in the induction of Tregs. The release of ATP from AML cells treated with chemotherapy leads to the upregulation of IDO1 in DCs. These DCs, in turn, are fully capable of inducing Tregs through the IDO1 pathway *in vitro* (64). Moreover, bone marrow mesenchymal stem cells (MSCs) derived from AML patients exhibited considerable upregulation of IDO and released heightened levels of PGD2. These factors collectively contributed to the expansion of Tregs (65, 66). PGD2 derived from MSCs engages the receptor CRTH2 on type 2 innate lymphoid cells (ILC2s) to promote the overproduction of IL-5, which specifically expands CD4+CD25+IL5Rα+ Tregs (66). Furthermore, experimental evidence has shown that the release of IFN-γ by AML cells *in vitro* triggers the upregulation of IDO expression in MSCs. Consequently, this upregulation contributes to the proliferation of Tregs (67, 68).

In AML patients, abnormally high levels of TNF-α secreted by Th17 cells promote Treg proliferation through the TNF-α receptor 2 (TNFR2) pathway expressed by Tregs (69). Additionally, AML blast cells also generate significant quantities of TNF-α, which have the potential to induce the proliferation of Tregs by upregulating the expression of TNFR2 and FOXP3 on T cells (70, 71). Further research has shown that TNF-α binding to TNFR2 activates the p38 MAPK signaling pathway, which upregulates the surface expression of TNFR2 and Foxp3 on Tregs, thereby driving their proliferation and expansion (72, 73). Azacitidine combined with lenalidomide or panobinostat therapy can reduce TNFR2+ Tregs *in vivo*, which may contribute to the maintenance of clinical remission (70, 74). Previous reports indicate that azacitidine promotes Treg expansion by hypomethylation of the CpG island associated with the promoter of the FOXP3 gene (75, 76). This potentially contradictory finding can be explained by several reasons. First, the combined drugs, lenalidomide or panobinostat, might reverse this effect of azacitidine. *In vitro* studies have provided evidence that lenalidomide can decrease the expression of FOXP3 and inhibit the expansion of Tregs mediated by IL-2 (77). Similarly, studies have shown that administering low doses of panobinostat can lead to a reduction in FOXP3 expression and Treg frequency (78). Additionally, azacitidine treatment indirectly decreases TNFR2+ Tregs by reducing the population of residual blast cells, as blast cells secrete TNF to stimulate Treg expansion (70, 79). Furthermore, within the AML microenvironment, Tregs express elevated levels of IL-35, which can further contribute to the expansion of Tregs themselves (80).

The expansion of Tregs is facilitated by the interaction between AML cells and Treg cells through receptor-ligand interactions. This includes the interaction of PD-L1 (B7-H1) on the surface of AML cells with PD-1 on Tregs, as well as the ICOSL/ICOS and CD200/CD200R interactions. The expression of PD-L1 on AML cells increases the population of PD1+ Tregs and suppresses anti-leukemia immunity (81, 82). The PD-L1/PD-1 pathway has been found to have a role in driving the conversion of naive T cells into FOXP3+ Tregs by antagonizing the Akt-mTOR signaling pathway (82). Blocking the PD-L1/PD-1 signaling pathway using anti-PD-L1 antibodies has been shown to reduce Treg production and delay the progression of AML in mouse models (83, 84). Han et al. revealed that AML cells possess the ability to express ICOSL, which interacts with ICOS on the surface of Tregs and fosters their proliferation. Through the utilization of an antibody targeting ICOSL, they successfully impeded the generation of ICOS-positive Tregs and effectively retarded the advancement of AML in a murine model (85). Studies have reported that elevated levels of CD200 expression in AML blasts promote the induction of Tregs (86, 87). Inhibition of the interaction between CD200 and its receptor CD200R has been shown to decrease the intensity of FOXP3 (87). Research has demonstrated that the GITR plays a role in promoting the differentiation and expansion of Tregs (88). Furthermore, studies have indicated that surface expression of GITR is increased in Treg of AML patients (45). However, further studies are needed to determine if and how GITR can promote Treg accumulation in AML. Zhou et al. found that Gal-9 defective mice were more resistant to AML cells than wild-type mice, which was associated with less Treg accumulation, hinting that Gal-9 on AML cells may be engaged in expansion of Treg (89). The Gal-9/TIM-3 signaling pathway has been found to contribute to excessive proliferation and activation of Treg cells in chronic lymphocytic leukemia (CLL) (90). Additional evidence is required to determine if a similar role exists in AML.

The expression of chemokine receptors has been demonstrated to play a role in the excessive accumulation of Tregs (91, 92). Specifically in AML, there is an increased presence of TNFR2+ Tregs, which exhibit a heightened capacity for migration towards the BM (74). Additionally, study has reported that the frequencies of Tregs in the BM are significantly higher compared to PB in the same patients with AML (49). *In vitro* research has also demonstrated that AML-induced DCs exert a significant chemotactic effect on Tregs, which may contribute to the accumulation of Tregs at the site of leukemia (93). Tregs in AML have been shown to display strong migration towards the BM due to their increased expression of the chemokine receptor CXCR4 (43). It has been found that blocking the CCL3-CCR1/CCR5 and CXCL12-CXCR4 axes can slow down AML progression by inhibiting the migration of Tregs into the leukemic hematopoietic microenvironment (94).

Additionally, the metabolic profile of Tregs provides them with a competitive advantage, indirectly promoting aggregation. The hypermetabolic state of tumor cells creates a low-glucose and lactate-rich microenvironment, which is unfavorable for immune effector cells. Tregs possess the ability to reprogram their metabolic profile by regulation of FOXP3, thereby conferring upon them a metabolic edge and enhanced adaptive capacity within this environment (95). In the B16-F10 melanoma mouse model, tumor-infiltrating Treg cells have the capability to utilize lactate as a source of energy to sustain their proliferation and functional activity in a glucose-deficient environment (96). Consistent with this, higher lactate concentrations were observed in BM of AML (97). Zhang et al. reaffirmed the contribution of AML cells to the lactate-rich TME, and then they employed the lactate transporter inhibitor Syrosingopine to reduce lactate production, which resulted in a reduction of Treg. Based on these findings, the researchers concluded that lactate produced by AML cells actively promotes the aggregation of Treg cells (44). Additionally, Tregs in AML displayed an enrichment of pathways linked to fatty acid metabolism, providing further evidence that Tregs have the capacity to enhance energy production through the utilization of fatty acids present in their surrounding environment (98).

### 2.3 The immunosuppressive mechanisms of Treg in the AML microenvironment

**Figure 2:**
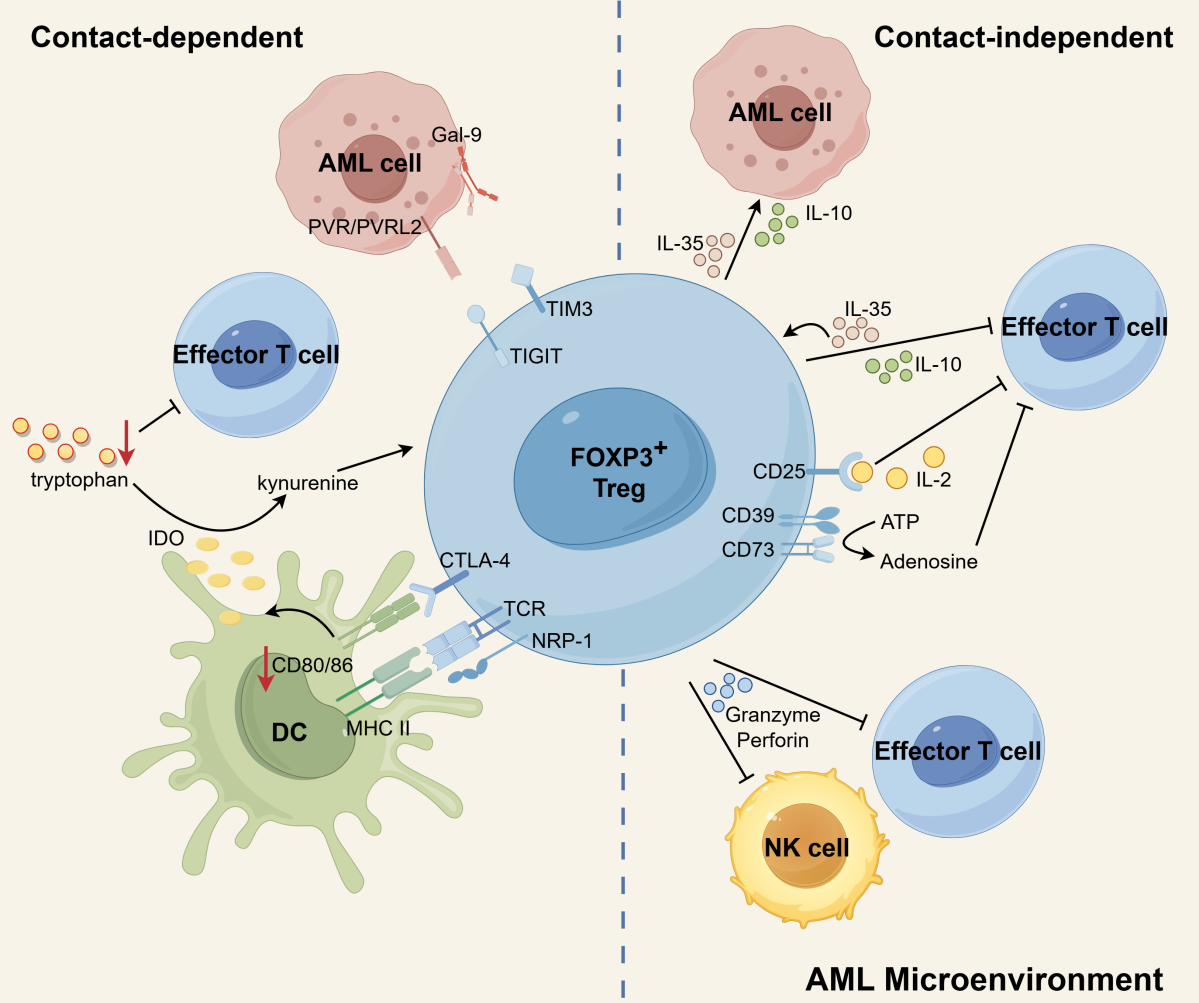
The immunosuppressive mechanisms of Tregs in AML microenvironment. CTLA-4 expressed by Tregs binds to CD80/86 on DCs, leading to inhibition of co-stimulation of Teffs, downregulation of CD80/86 on DCs, and elevated expression of IDO in DCs. By degrading tryptophan to kynurenines, IDO contributes to the induction of Tregs and the suppression of T-cell responses. Additionally, NRP1 prolongs the MHC-II molecule-dependent interactions between Tregs and DCs, which effectively restricts the recruitment of MHC-II peptides to immune synapses, ultimately inhibiting immune responses. Treg-derived IL-10 diminishes anti-leukemia immunity by suppressing the activity of Teffs. IL-35 released by Treg can suppress Teff functions and proliferation while also expanding a population of inducible Tregs. IL-10 and IL-35 also stimulate the proliferation of AML blasts. Additionally, Tregs can induce cell death in NK and Teff cells by utilizing granzyme and perforin. CD25, expressed on Tregs, allows for continuous uptake of IL-2, leading to the cytokine deprivation-induced apoptosis of Teff cells. Tregs express membrane surface enzymes CD39 and CD73, which can hydrolyze ATP to generate adenosine. Adenosine, in turn, inhibits cytokine production and proliferation of Teff cells, further contributing to the suppressive function of Tregs. In addition, the possible existence of TIGIT-PVR/PVRL2 and TIM3-Gal9 signaling pathways between Tregs and AML cells may contribute to a propensity for leukemia progression. Schematic figure was drawn by Figdraw (www.figdraw.com).

Tregs play a pivotal role in the inhibition of immune effector cells, ultimately leading to the impairment of anti-leukemia immune responses in AML. Tregs achieve this immunosuppressive effect through two ways: cell-to-cell contact and contact-independent pathways (**Figure 2**). The contact-dependent mechanism primarily involves intricate receptor-ligand interactions between cells, while the contact-independent mechanism predominantly relies on cytokine secretion and other non-secretory means. Subsequently, this section will provide an elaborate elucidation of how Treg cells effectively suppress immune effector cells in AML by employing these two mechanisms.

#### 2.3.1 Contact-dependent mechanism

Contact-dependent immunosuppression heavily relies on the interaction between surface molecules expressed by Tregs and other cells. Notably, investigations have revealed that Tregs in AML enhance the expression of specific suppressive surface molecules. In particular, Tregs derived from individuals with AML have demonstrated elevated levels of CTLA-4 expression (45, 50). The expression of CTLA-4 by Tregs hinders the co-stimulation of effector T cells (Teffs) by outcompeting CD28 for binding to CD80/86 on antigen-presenting cells (APCs) (99). Additionally, CTLA-4 on Tregs downregulates the expression of CD80/86 on DCs, thereby impeding the activation of Teffs (99, 100). Furthermore, the interaction between CTLA-4 and CD80/86 triggers an upregulation of IDO in DCs (62, 101). IDO, in turn, degrades tryptophan within the microenvironment, leading to the suppression of T-cell responses (102) and the generation of Tregs (55). In acute leukemia patients, there is an observed increase in the expression of NRP-1 on Tregs. Interestingly, the introduction of exogenous Sema3A, which serves as a ligand for NRP-1, can effectively downregulate NRP-1 expression on Tregs and facilitate the apoptosis of leukemia cells (103). Notably, NRP-1 is highly expressed on intratumoral Tregs (104), and enables prolonged interactions between Tregs and DCs that are dependent on MHC-II molecules. This, in turn, restricts the recruitment of MHC-II peptide complexes to immune synapses, ultimately impeding immune responses (105).

There are some studies implicating that Tregs may interact with AML cells through TIGIT and TIM-3 to help them escape immune surveillance. TIGIT, as a co-inhibitory receptor, was found to be ubiquitously expressed on the Tregs in AML (44, 53). The activation of TIGIT signaling leads to the upregulation of suppressive genes (such as *Pdcd1*, *IL10*, *Prf1*, and *Havcr2*) in TIGIT-positive Tregs, resulting in the manifestation of a highly activated suppressive phenotype (106). Stamm et al. conducted a study demonstrating that AML cell lines and patient samples exhibit high expression levels of the TIGIT ligands, PVR and PVRL2, which correlates with a poor prognosis. They further revealed that blocking PVR/PVRL2 on AML cells or inhibiting TIGIT on immune cells enhances the anti-leukemic effects *in vitro* (107). Moreover, TIGIT+ Tregs were found to upregulate the expression of the co-inhibitory receptor TIM-3, suggesting a collaborative suppression of antitumor responses by TIM-3 and TIGIT (106). Indeed, it was observed that TIM-3+ Treg cells significantly increased in *de novo* AML patients (108). High levels of Gal-9 (the ligand of TIM-3) were also observed on leukemia blasts in AML samples (109, 110). Interestingly, TIM-3 is also expressed on leukemic stem cells in AML (111, 112), and even Gal-9 has been shown to be expressed on activated Treg (113). These studies illustrate that Gal-9 and TIM-3 may engage in complex interactions within the AML microenvironment.

#### 2.3.2 Contact-independent mechanism

Cytokines, granzyme and perforin are involved in a contact-independent mechanism (40, 80, 114). Newly diagnosed AML patients have been found to exhibit heightened levels of Treg-associated cytokines, specifically IL-10 and IL-35 (115). The immunosuppressive factor IL-10, derived from Tregs, plays a crucial role in diminishing anti-tumor immune responses by suppressing the activity of Teffs and APCs (116). IL-35 has the ability to suppress the functions and proliferation of Teffs, while simultaneously promoting the expansion of inducible Tregs (117, 118). In the AML microenvironment, both IL-10 and IL-35 not only exert inhibitory effects on immune cells but also contribute to the stimulation of AML blast proliferation. The highly expressed cytokine IL-10 by Tregs has been shown to enhance the stemness of AML cells by activating the PI3K/AKT signaling pathway. In AML/ETO c-kit^mut^ (A/Ec) leukemia mice, blocking the IL10/IL10R/PI3K/AKT signaling pathway extended their survival and significantly reduced the stemness of A/Ec leukemia cells. Furthermore, a positive correlation was found between the proportion of Tregs and leukemia stem cells (LSCs) in patient samples. AML patients with high Treg infiltration also exhibited stronger activation of the PI3K/AKT pathway in CD34+ primary AML cells (119). Additionally, IL-35 has been shown to directly promote the proliferation of AML blasts and inhibit their apoptosis (80). The expression of perforin and granzyme B is upregulated in Tregs of patients with AML compared to healthy individuals. Additionally, Tregs in AML patients have been shown to exert immunosuppressive effects by utilizing perforin and granzyme B (45). Tregs have the ability to induce apoptosis in natural killer (NK) cells and CD8+ T cells by utilizing granzyme B and perforin. Research indicates that mice lacking granzyme B show improved efficacy in clearing AML cells in comparison to mice with intact granzyme B functionality. Moreover, when wild-type Treg cells are introduced into granzyme B-deficient mice, there is a discernible suppression of AML clearance (114).

In addition to the secretion, the uptake and enzymatic hydrolysis of factors from the microenvironment also occur independently of contact. The constitutive expression of CD25, which represents high affinity IL-2 receptors, allows Treg cells to continually absorb IL-2. This uptake of IL-2 leads to cytokine deprivation-induced apoptosis of Teff cells (120). Tregs constitutively express the membrane surface enzymes CD39 and CD73. These enzymes have the ability to hydrolyze ATP or ADP, resulting in the production of adenosine. Consequently, the levels of adenosine in the microenvironment are elevated. Adenosine, in turn, interacts with the adenosine receptor A2A on the surface of Teff cells, leading to the inhibition of cytokine production and proliferation (33). Indeed, study has shown that CD39 and CD73 are expressed on CD4+CD25^high^ Tregs isolated from patients with AML. Interestingly, Tregs obtained from AML patients have been shown to have a higher ability to hydrolyze ATP into adenosine compared to Tregs from healthy individuals (45).

### 2.4 Potential immunotherapy strategies targeting Treg in AML

Currently, immunotherapy for AML targeting Tregs represents an extremely promising treatment, with a main focus on reducing the number of Tregs (**Table 2**). Evidence suggests that the downregulation of Tregs coincides with an increase in antileukemic reactivity (121). The combination therapy of Ara-C, a CXCR4 inhibitor, and PD-L1 mAb has been shown to enhance the eradication of leukemic myeloid blast cells by effectively suppressing Tregs (122). In mouse models, it has been shown that the depletion of Tregs using anti-CD25 antibodies prior to DC vaccination against AML significantly enhances the immune response against leukemia. This approach facilitates the development of robust and long-lasting immune responses (123). The depletion of Tregs using anti-CD25 antibody (124) or interleukin-2 diphtheria toxin (IL-2DT) (NCT01106950) (125) prior to IL-2 administration has demonstrated enhanced antileukemic effects mediated by NK cells. Similarly, IL-2DT can eliminate Tregs, increasing the quantity of transferred cytotoxic T lymphocytes (CTL) at AML disease sites and reducing tumor burden (126). Clinical trials (NCT00675831, NCT00987987) have shown that a donor lymphocyte infusion depleted of CD25+ Tregs can lead to enhanced anti-tumor efficacy in patients with hematologic malignancies who have experienced relapse after undergoing allo-HSCT (127, 128). The safety and efficacy of the combined treatment strategy of infusion of Treg-depleted T lymphocytes and WT1 antigen-specific cancer immunotherapeutic in patients with WT1-positive AML are under evaluation (NCT01513109). Various targets highly expressed on Treg cells, including LAG3, TIM3, VISTA, TIGIT, OX40, ICOS, and chemokine receptors such as CCR4, CCR5, and CCR8, have been suggested as potential targets for eliminating Treg cells (116). These studies suggest that reducing the population of Treg cells may hold therapeutic benefits in the treatment of AML.

**Table 2:** AML treatment through reducing Treg numbers.

Target	Treatment	Study IDs	Research stage	Clinical outcomes	References
CD25	anti-CD25 Ab	——	preclinical phase	——	(123, 124)
	IL-2DT	NCT01106950	Phase II (Terminated)	Depletion of host Tregs with IL2DT improves efficacy of haploidentical NK cell therapy for refractory AML.	(125)
		——	preclinical phase	——	(126)
——	Treg-depleted donor lymphocytes infusion	NCT00675831	Phase I (Completed)	Treg-depleted donor lymphocytes infusion was associated with a better response rate and improved event-free survival.	(127)
		NCT00987987	Phase I/II (Completed)	Treg-depleted donor lymphocyte infusion safely induces graft-versus-host/tumor effects in alloreactivity-resistant patients.	(128)
		NCT01513109	Phase I/II (Unknown status)	——	——

## 3 The other usual one: myeloid-derived suppressor cell

### 3.1 The phenotype of MDSC

The TME impedes the normal differentiation of hematopoietic stem cells, resulting in the emergence of a subset of immature and heterogeneous myeloid cells called MDSCs (129). MDSCs can be broadly classified into two main categories: monocytic MDSCs (M-MDSCs) and polymorphonuclear MDSCs (PMN-MDSCs). M-MDSCs are characterized as Lin−(CD3, CD19, CD56)CD11b+CD15−CD14+HLA-DR^low/−^, while PMN-MDSCs are defined as Lin−CD11b+CD15+CD14−CD66b+HLA-DR^low/−^ (130, 131). M-MDSCs exhibit phenotypic and morphological similarities to monocytes, while PMN-MDSCs share closer resemblance to neutrophils (129). In humans, M-MDSCs can be distinguished from monocytes by the absence of MHC class II molecules, and the population of PMN-MDSCs can be identified using LOX-1 as a marker to differentiate them from neutrophils (132, 133). PMN-MDSCs comprise the majority of MDSCs, accounting for more than 75% of the population, whereas M-MDSCs make up only 10-20% (133). However, it is important to highlight that M-MDSCs possess a higher immunosuppressive potential compared to PMN-MDSCs (133, 134). In recent years, researchers have identified a small population of human bone marrow progenitor and precursor cells that exhibit colony-forming activity. These cells, known as early myeloid-derived suppressor cells (eMDSCs), are characterized by their labeling as Lin−HLA-DR^low/−^CD11b+CD14−CD15−CD33+ (131).

### 3.2 MDSC accumulation and its mechanisms in AML

**Substantial evidence suggests that MDSCs are expanded in AML and significantly contributes to poor prognosis.** Specifically, in C57BL/6 mice engrafted with TIB-49 AML, an expansion of CD11b+Gr11+ MDSCs was observed in both the BM and spleen (135). Clinical studies have demonstrated that adult patients with AML exhibit significantly elevated frequency of MDSCs in their BM. These MDSCs are identified by CD33^high^CD11b+HLA-DR^low/neg^. Importantly, it has been observed that the proportion of MDSCs decreased after patients achieve CR. Additionally, the frequency of MDSCs is positively correlated with minimal residual disease (MRD) levels, suggesting that these cells may impact the clinical course and prognosis of AML (136). Studies have provided evidence that circulating M-MDSCs are increased in individuals with AML. Moreover, the presence of elevated M-MDSC percentage has been associated with a low CR rate, a high relapse/refractory rate, and poor long-term survival in AML patients (137–139). In a monocentric prospective study on AML, two independent negative prognostic indicators for overall survival were identified: an initial peripheral percentage of M-MDSCs exceeding 0.55% of leukocytes at the time of diagnosis, and a subsequent decrease in the percentage of M-MDSCs following induction therapy (140). Research conducted by Hyun et al. demonstrated that AML patients with a heightened frequency of MDSC-like blasts, characterized by elevated levels of ARG-1 and iNOS, exhibited the ability to suppress T cell proliferation, thereby contributing to an unfavorable prognosis (141).

**Figure 3:**
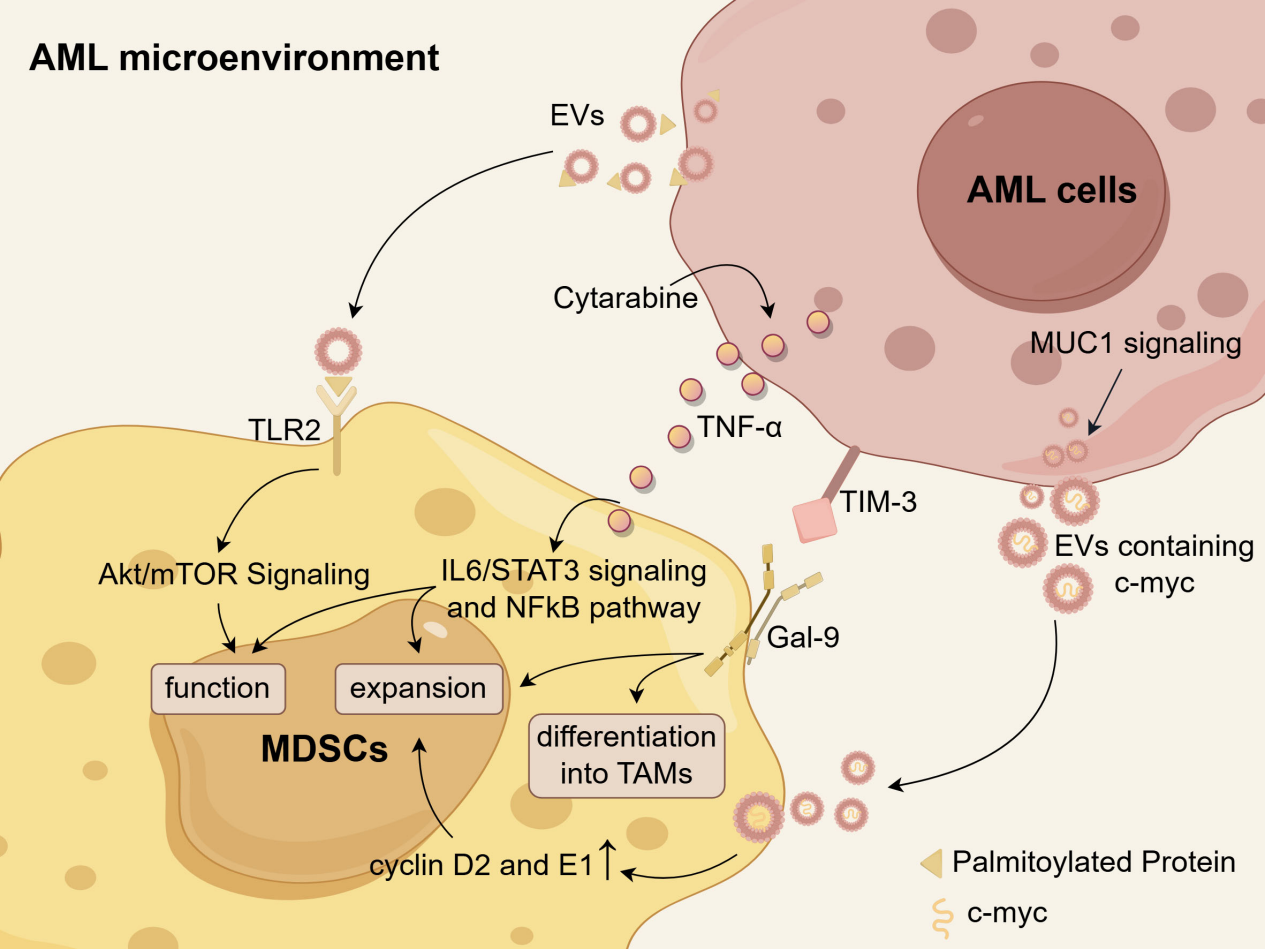
The mechanisms of MDSC accumulation in the AML microenvironment. Palmitoylated proteins present on the surface of AML-derived EVs activate TLR2, triggering the Akt/mTOR-dependent induction of MDSCs. Cytarabine-induced TNF-α secretion from AML cells leads to an expansion of MDSCs and enhances their functions and survival by activating IL-6/STAT3 signaling and NFκB pathways. AML cells secrete EVs containing c-myc in a MUC1-dependent manner, which facilitates MDSC proliferation through upregulation of cyclin D2 and E1. There is a hypothesis that the Tim-3/Gal-9 pathway may promote the expansion of MDSCs and their differentiation into TAMs in AML. Schematic figure was drawn by Figdraw (www.figdraw.com).

Extensive research has been conducted to investigate the mechanisms of MDSC accumulation. AML-derived EVs are an important factor contributing to the accumulation of MDSCs in AML. Specifically, palmitoylated proteins present on the surface of AML-EVs activate Toll-like receptor 2 (TLR2) of monocytes and trigger MDSC induction controlled by Akt/mTOR signaling pathway (142). Therefore, targeting protein palmitoylation could serve as a potential approach to disrupt the differentiation of MDSCs. Additionally, AML cells employ a MUC1-dependent mechanism to secrete EVs containing c-myc, when co-cultured with MDSCs. The presence of these EVs subsequently prompts the upregulation of cyclin D2 and cyclin E1 in MDSCs, suggesting that the c-myc-containing EVs potentially enhance MDSC proliferation (135). Cytarabine (Ara-C) treatment prompted AML cells to express and secret TNF-α, which subsequently facilitated the expansion of MDSCs and enhanced their function and survival through activating IL-6/STAT3 and NFκB pathways (143). Additionally, Gao et al. proposed the hypothesis that TIM-3 on AML stem cells interacts with Gal-9 on MDSCs, thereby promoting the expansion of MDSCs and their differentiation into tumor-associated macrophages (TAMs) (144). However, further research is necessary to validate this hypothesis. Theoretically, if the increase in MDSCs could be inhibited based on these mechanisms, it may offer a potential rescue strategy for AML patients. The mechanisms underlying MDSC accumulation within the AML microenvironment are illustrated in **Figure 3**.

### 3.3 The immunosuppressive mechanisms of MDSC in the AML microenvironment

MDSCs exhibit immunosuppressive activities that hinder effective anti-leukemic immune responses. MDSCs accumulated in the PB of AML patients exhibit high expression of VISTA, which is thought to be associated with the suppression of the T-cell response. Evidence suggests that VISTA exerts an inhibitory effect on the anti-leukemia T-cell response, as demonstrated by the effective reduction of MDSC-mediated CD8+ T-cell inhibition in AML following VISTA knockdown using specific siRNA (145). However, the precise mechanisms by which MDSCs operate within the AML microenvironment remain unclear at present, underscoring the urgent need for a more detailed investigation of their functional roles.

### 3.4 Potential immunotherapy strategies targeting MDSC in AML

Targeted intervention of MDSCs has the potential to attenuate their immunosuppressive capabilities and strengthen the immune response against leukemia. In an AML mouse model, Hwang et al. demonstrated that a triple combination therapy consisting of Ara-C, a CXCR4 inhibitor, and a PD-L1 mAb resulted in a significant reduction of MDSCs and a potent eradication of leukemic myeloid blast cells (122). In addition, a clinical trial (NCT01347996) demonstrated a notable decrease in peripheral M-MDSCs among AML patients treated with histamine dihydrochloride (HDC) and low-dose IL-2 for relapse prevention, heralding a promising clinical outcome (146). Given the prevalent expression of CD33 on MDSCs, CD33 is frequently employed as a target of MDSCs (147). The CD33/CD3-bispecific T-cell engaging (BiTE^®^) antibody (AMG 330) exhibited notable efficacy in combating leukemia by specifically targeting CD33+ MDSCs in AML (148). A multicenter clinical trial (NCT03214666) is currently underway to investigate the potential of CD16/IL-15/CD33 tri-specific killer cell engager (GTB-3550 TriKE^®^) in targeting CD33+ MDSCs. The 123NL CAR-T therapy, which has been designed to target CD123 and NKG2DL, has demonstrated the ability to effectively eliminate M-MDSCs in AML (149). In a murine AML model, treatment with the hypomethylating agent guadecitabine (SGI-110) has been shown to reduce the MDSC burden, subsequently resulting in an increase proportion of functionally active leukemia-specific T cells (150). These studies suggest that targeted decrease of MDSCs is advantageous for the AML treatment. Immunotherapy strategies targeting MDSC in AML are summarized in **Table 3**.

**Table 3:** AML treatment through targeting MDSC.

Target	Treatment	Study IDs	Research stage	Clinical outcomes	References
CD33	CD33/CD3-bispecific T-cell engaging (BiTE^®^) antibody (AMG 330)	——	preclinical phase	——	(148)
	CD16/IL-15/CD33 tri-specific killer cell engager (GTB-3550 TriKE^®^)	NCT03214666	Phase I/II (Terminated)	Study terminated prematurely with no analyzable results.	——
CD123 and NKG2DL	123NL CAR-T	——	preclinical phase	——	(149)
——	guadecitabine (SGI-110)	——	preclinical phase	——	(150)
——	Combination therapy with Ara-C, CXCR4 inhibitor and PD-L1 mAb	——	preclinical phase	——	(122)
——	HDC and low-dose IL-2	NCT01347996	Phase IV (Completed)	Peripheral M-MDSCs were reduced during HDC/IL-2 therapy, heralding favorable clinical outcome.	(146)

## 4 The other developing one: leukemia-associated macrophage

### 4.1 The phenotype of LAM

Tumor-associated macrophages within the leukemia microenvironment, specifically referred to as LAMs, have been documented to play a significant role in the progression of leukemia. Macrophages can undergo polarization from the M0 state into classically activated (M1) macrophages, which demonstrate anti-leukemic and immunostimulatory capabilities, or alternatively activated (M2) macrophages, which exhibit pro-leukemic and immunosuppressive characteristics (151, 152). LAMs share functional characteristics with both M1- and M2-like macrophages. However, they predominantly align with the pro-leukemic properties of M2 macrophages (151, 153). M2 macrophages are characterized by the expression of surface markers such as CD163, CD206, and the M-CSF receptor CD115. Additionally, they secrete arginase II (Arg2), chitinase-3-like protein 1 (CHI3L1/YKL-40), and the anti-inflammatory cytokines IL-10 and TGF-β, which contribute to their immunosuppressive and tumor-promoting roles (154).

### 4.2 LAM accumulation and its mechanisms in AML

The expansion of M2-like LAMs in AML is a contributor to a negative prognosis. Al-Matary et al. demonstrated that M2-like macrophages were elevated in the BM of AML patients and mice (155). It has been observed that more M2-like LAMs are associated with a worse prognosis in AML patients (156, 157). Tian et al. found that the proportion and number of LAMs were higher in patients with refractory AML than in those who achieved CR (156). Consistent with this finding, a study by Brauneck et al. demonstrated an increased frequency of BM-infiltrating immunosuppressive M2 macrophages expressing TIGIT, TIM-3, and LAG-3 in patients with newly diagnosed and relapsed AML (157). Xu et al. reaffirmed that M2-like LAMs, characterized by CD206 positivity, are predominantly enriched within the AML microenvironment, and a high infiltration of M2 macrophages is correlated with adverse clinical outcomes (158). Patients with AML exhibiting elevated levels of CD163 transcripts demonstrated a diminished likelihood of survival (159). This finding aligns with the results reported by Guo et al. through single-cell RNA sequencing, which identified a specific monocyte/macrophage cluster characterized by high CD163 expression that correlates with a reduced probability of survival in AML patients (160).

**Figure 4:**
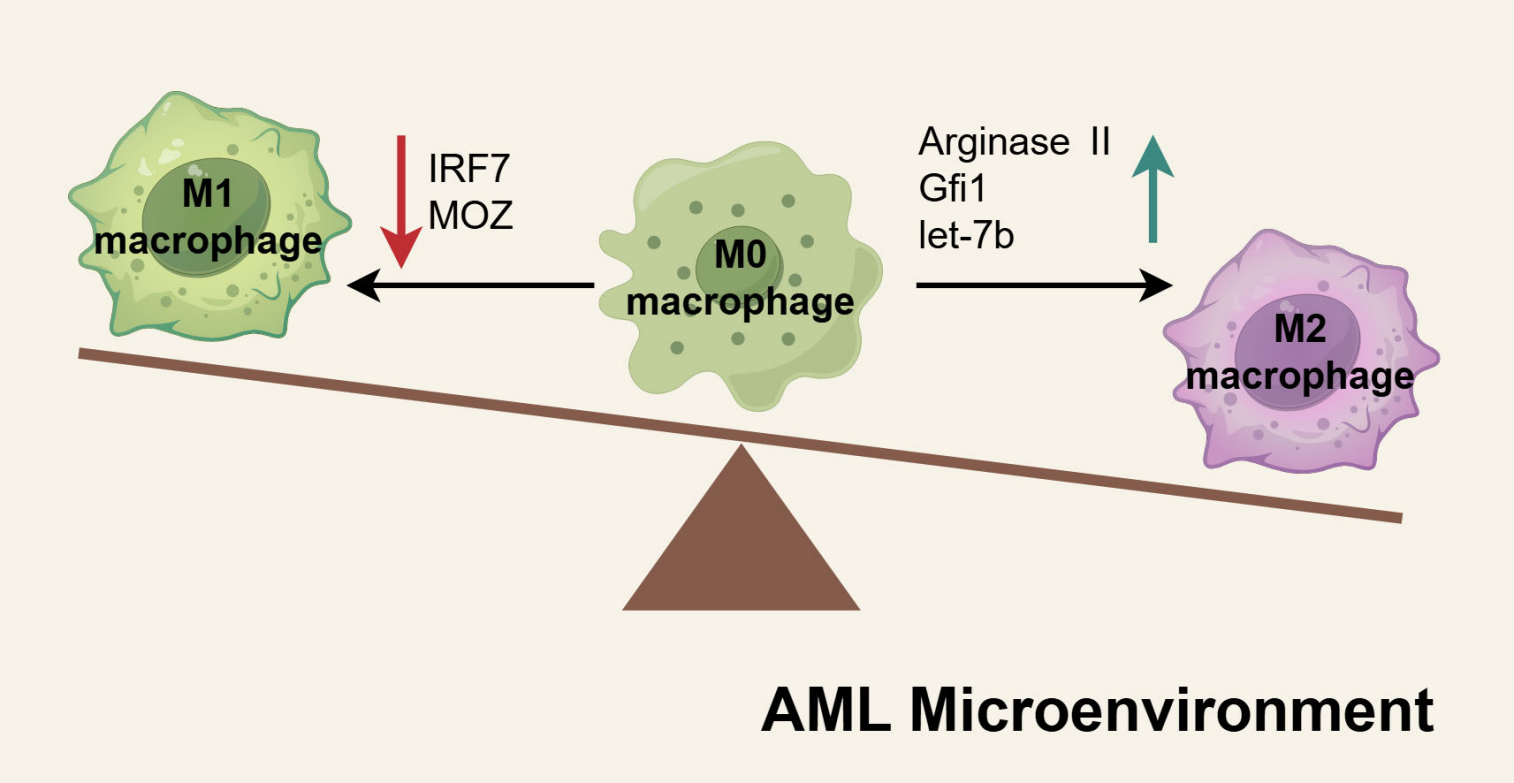
The mechanisms of M2-like LAMs accumulated in AML microenvironment. In the AML microenvironment, the factors regulating M1 or M2 macrophage polarization are dysregulated, creating an imbalance in macrophage differentiation. Pro-M1 factors such as IRF7 and MOZ are downregulated in AML macrophages, resulting in diminished M1 activation. Conversely, elevated levels of pro-M2 factors, including arginase II, Gfi1, and let-7b, drive increased polarization toward the M2 phenotype. These shifts culminate in the accumulation of M2-like macrophages within the AML microenvironment, fostering an immunosuppressive milieu that supports leukemia progression. Schematic figure was drawn by Figdraw (www.figdraw.com).

The mechanisms underlying the increase of M2-like LAMs has been comprehensively investigated. There is increasing evidence that the factors influencing M1 and M2 characteristics are imbalanced within the AML microenvironment, resulting in a greater accumulation of M2-like LAMs (**Figure 4**). Using *in vitro* and *in vivo* models, Mussai et al. provided the first reports demonstrating that the secretion of arginase II by AML blasts induces the polarization of monocytes into an immunosuppressive M2-like phenotype, marked by the increased expression of CD206 (161). The transcription factor Gfi1 expression was about two-fold upregulated in LAMs of AML compared to non-leukemic macrophages, and it promote the polarization of macrophages to a leukemia-supporting state (155). Recently, Tian et al. identified let-7b as a potential aberrant gene implicated in conferring M2-like characteristics and demonstrated its significant upregulation in LAMs from refractory AML mice. Knockdown of let-7b in LAMs was shown to suppress AML progression by reprogramming LAMs toward an M1-like phenotype, mediated through the activation of the Toll-like receptor and NF-κB signaling pathways (156). Jiang et al. discovered that low levels of MOZ correlate with poor prognosis in AML. They observed that the loss of MOZ led to reduced M1 activation in macrophages and heightened resistance to chemotherapeutic agents (162). Similarly, IRF7, a key contributor to M1 polarization, was found to be underexpressed in the more immunosuppressive phenotype of spleen-derived LAMs. IRF7 promotes M1 characteristics by activating the SAPK/JNK pathway in macrophages, and stimulation of this pathway was shown to significantly extend the survival duration of AML mice (159).

### 4.3 The immunosuppressive mechanisms of LAM in the AML microenvironment

The interplay between LAMs and AML blasts enhances AML cell survival. M2-like macrophages secrete soluble factors such as CCL2 and CXCL8, which activate pro-survival pathways and suppress apoptosis in leukemic blasts (151). Williams et al. show that M2-like macrophages protect the U937 and THP-1 AML cell lines against daunorubicin-induced apoptosis (163). While the role of TAMs in solid tumors has been extensively studied (164), the significance of LAMs in leukemia has only recently gained attention due to the unique and heterogeneous nature of leukemic microenvironments. Overall, the precise mechanisms by which LAMs influence AML remain poorly understood.

### 4.4 Potential immunotherapy strategies targeting LAM in AML

To counteract the immunosuppressive and leukemia-promoting effects mediated by M2-like LAMs in AML, current effective strategies primarily focus on depletion and reprogramming. In a mouse model of MLL-AF9-driven AML, Keech et al. demonstrated that targeted depletion of CD169+/SIGLEC1+ macrophages via diphtheria toxin injection significantly extended median survival in mice treated with cytarabine and doxorubicin (165). Furthermore, the 123NL CAR-T therapy designed to target CD123 and NKG2DL, has proven effective in eliminating M2 macrophages in AML (149). Experimental evidence indicates that knockdown of let-7b in LAMs causes M1-like polarization, thereby significantly inhibiting the progression of AML in a mouse model driven by MLL-AF9 (156). Additionally, Liu et al. revealed that chenodeoxycholic acid (CDCA) inhibited the polarization of M2-like LAMs and curtailed their proliferation-promoting effects on AML cells (166). Moreover, *in vitro* blockade of TIGIT reprograms M2 LAMs toward an M1 phenotype and enhances anti-CD47-mediated phagocytosis of AML cells (157). Immunotherapy strategies targeting LAMs in AML are comprehensively summarized in **Table 4**.

**Table 4:** AML treatment through targeting LAM.

Therapeutic strategies	Treatment	Research stage	References
Depletion of LAMs	specific depletion of CD169+ macrophages	preclinical phase	(165)
	123NL CAR-T therapy	preclinical phase	(149)
Reprogramming LAMs	knockdown of let-7b	preclinical phase	(156)
	chenodeoxycholic acid (CDCA)	preclinical phase	(166)
	blockade of TIGIT	preclinical phase	(157)

## 5 The other emerging one: regulatory B cell

As early as the 1970s, researchers proposed that certain B cells could exert immunosuppressive function by secreting inhibitory cytokines (167). In 2002, Mizoguchi identified a subset of B cells characterized by up-regulation of CD1d in the mesenteric lymph nodes of intestinal inflammation murine models, which inhibited the progression of enteritis by producing IL-10, and defined this group of B cells with immunomodulatory functions as regulatory B cells (Breg) (168). Currently, the origin and development of Breg cells are poorly understood. It is widely accepted that immature and mature B cells, as well as plasmablasts, can differentiate into Breg cells under appropriate stimulation and timing, resulting in a heterogeneous Breg population (169). Several different subtypes of Breg cells have been identified in humans and mice, though specific biomarkers for Breg cell activation have yet to be established (169, 170). The phenotypes of major Breg subsets are summarized in **Table 5**. The most well-characterized human Breg phenotypes include CD19+CD24^high^CD38^high^ (171) and CD19+CD24^high^CD27+ (172).

**Table 5:** Phenotypes of Breg subsets in humans and mice.

Breg type	Human	Mouse	Reference
B10 cells	CD24^hi^CD27+	CD19+CD5+CD1d^hi^	(172, 232)
T2-MZP cells	——	CD19+CD21^hi^CD23^hi^CD24^hi^	(233)
Plasma cells	——	CD138+MHC-11^lo^B220+	(234)
MZ cells	——	CD19+CD21^hi^CD23−	(235)
Tim-1+ B cells	——	Tim-1+CD19+	(236)
Plasmablasts	CD19+CD27^int^CD38+	CD138+CD44^hi^	(237)
Immature cells	CD19+CD24^hi^CD38^hi^	——	(171)
Br1 cells	CD19+CD25^hi^CD71^hi^	——	(238)
GrB+ B cell	CD19+CD38+CD1d+IgM+CD147+	——	(239)
CD9+	CD19+CD9+	CD19+CD9+	(240)

The absence of definitive biomarkers for Breg cells considerably impedes research advancements, particularly in the context of AML, where investigations remain markedly constrained. Wan et al. demonstrated a significant elevation in the proportion of CD19+CD24^high^CD38^high^ Breg cells within the BM of AML patients (43). This aligns with the findings of Lv et al., who observed an elevated frequency of Breg cells in both PB and BM of AML patients compared to healthy controls, and this increased frequency was associated with a shorter overall survival (173). However, a subsequent study by Dong et al. found that patients with newly diagnosed AML exhibited a significantly lower Breg frequency in PB than healthy controls (174). Interestingly, all three studies utilized CD19, CD24, and CD38 as markers to define Breg cells, yet their results exhibited notable inconsistencies. Wan’s study enrolled 45 patients, Lv’s included 46, and Dong’s involved 40. This divergence may be attributed to their relatively limited sample sizes. Furthermore, the inclusion of samples from both PB and BM sources could have introduced variability, potentially compromising the accuracy of the findings. To resolve this controversy, more extensive, well-replicated studies are imperative. Shi et al. demonstrated that PD-L1 expression was elevated on Breg cells from AML patients, with higher PD-L1 levels correlating with poorer prognosis (175). Research on Breg in AML is indeed quite scarce, underscoring both the significance and urgency of this investigative focus.

## 6 The other newly identified one: leukemia-associated neutrophils

Within the microenvironment of AML, leukemia/tumor-associated neutrophils (LANs/TANs) have emerged as a critical cellular component with increasingly recognized pathophysiological significance.

TANs are neutrophils recruited to tumor sites via chemokines (including CXCL1, CXCL2, and IL-8) secreted by tumor cells and stromal cells. Functionally, TANs can be polarized into anti-tumor N1 and pro-tumor N2 phenotypes. N1-type TANs are characterized by high expression of ICAM-1 and CD95, exerting anti-tumor effects through the release of ROS and cytokines such as IFN-γ. In contrast, N2-type TANs exhibit elevated expression of CCL2, IL-8, and ARG1, promoting tumor progression via angiogenesis induction, extracellular matrix remodeling, and immunosuppressive microenvironment formation (176).

In AML, LANs’ functional role is unclear, but an FGFR1-driven murine model revealed leukemogenesis polarizes neutrophils into six subsets (notably Ly6g+ and Camk1d+), which upregulate MMP8/9 to migrate from bone marrow to blood and differentiate into PMN-MDSCs; MMP inhibition with Ilomastat blocked migration and improved survival, while clinical data linked high MMP8 to poor AML outcomes, highlighting MMP8 as a potential therapeutic target to disrupt immune evasion (177).

## 7 Discussions and future prospects

The immunosuppressive role in AML orchestrated by immunosuppressive cells persists as a critical impediment to eliciting a robust anti-leukemic immune response. Despite significant advancements in understanding these cells, the development of viable therapeutic strategies remains an ongoing challenge, requiring further innovation and exploration.

### 7.1 Tregs in AML: current understanding and future directions

While research on Tregs in AML remains challenging, the function mechanisms of Treg in the solid tumor have been more clearly elucidated. Tumor-infiltrating Tregs heightened activation and potent immunosuppressive capabilities, characterized by elevated expression of LAG-3, LFA-1, TGF-β, EVs, and others (116). LAG-3 expressed on the surface of Treg could bind with a high affinity to MHC class II molecules on the surface of DCs, effectively inhibiting the maturation and immunostimulatory capacity of DCs (178). Treg-expressed LFA-1 has been shown to be involved in downregulating CD80/86 on DCs (179). TGF-β produced by intratumoral Tregs directly inhibited proliferation and differentiation of immunocompetent cells (180). Contrary to observations in solid tumors, AML demonstrates distinct TGF-β dynamics, with studies reporting either unchanged or reduced TGF-β levels in AML patients (115, 174). The underlying mechanisms for this differential expression remain unclear and warrant further investigation. Through gap junctions, Tregs deliver substantial quantities of cAMP to Teff cells, inducing metabolic interference that culminates in Teff suppression and apoptosis (181, 182). Additionally, recent studies have identified a novel suppression mechanism involving Treg-derived EVs. These EVs serve as bioactive carriers of proteins, lipids, and nucleic acids, orchestrating intercellular communication networks and modulating anti-tumor immunity (183). It was demonstrated that EVs derived from natural CD8+CD25+ Treg cells, containing LAMP-1 and CD9, were observed to significantly inhibit CTL responses and anti-tumor immunity in a B16 melanoma model (184). While established mechanisms of Treg-mediated immunosuppression in solid tumors provide a valuable framework for investigating their role in AML, critical distinctions must be acknowledged. The TME exhibits remarkable complexity, with Treg populations demonstrating substantial functional and phenotypic heterogeneity that varies significantly across different tumor subtypes (185). Additionally, emerging evidence suggests that Tregs may develop distinct functional properties within the unique leukemic microenvironment. TIGIT was ubiquitously expressed on the Tregs in AML (44, 53), and its ligands PVR and PVRL2 have been reported to be highly expressed on AML cell lines and patient samples (107). Moreover, antibody blockade of PVR or PVRL2 on AML cell lines or primary AML cells or TIGIT blockade on immune cells could enhance the anti-leukemic effects (107). It is possible that TIGIT on Treg cells may engage with PVR/PVRL2 on AML cells, thereby protecting leukemic cells from immune attack. However, there is no direct evidence so far. Further research is needed to determine the function of these molecules. A marked increase in TIM-3+ Treg cell populations was observed among *de novo* AML cases (108). Previous studies have reported that TIM-3+ Tregs in CLL drive immunosuppression via its ligand soluble Gal-9 (90). High levels of Gal-9 expression were also observed on blasts in primary AML samples (109, 110). Whether a similar situation exists in AML requires further study. Interestingly, TIM-3 is also expressed on AML stem cells (111, 112), and even Gal-9 has been shown to be expressed on activated Treg (113). These studies illustrate that the interaction between Gal-9 and TIM-3 in the AML immune microenvironment is complex and needs further exploration.

In AML treatment strategies, therapeutic depletion of Tregs can potentiate antileukemic immunity and improve clinical outcomes. However, any pharmacological approaches to reduce Treg frequency should be carefully optimized to mitigate potential adverse effects, including autoimmune reactions or uncontrolled inflammatory responses resulting from Treg dysregulation. Given the pivotal role of Treg homeostasis, targeting the molecular mechanisms underlying their accumulation represents a promising therapeutic avenue. Disrupting these pathways—such as with the IDO inhibitor 1-MT, which has demonstrated efficacy in suppressing Treg expansion—could offer a novel and clinically viable strategy for AML immunotherapy (56, 57). To facilitate clinical translation, rigorous evaluation of therapeutic feasibility remains essential, alongside the development of novel agents with optimized efficacy and safety profiles. Alternatively, attenuating Treg functionality represents a viable strategy to counteract the immunosuppressive AML microenvironment. OX40 activation has been shown to diminish Treg-mediated immunosuppression (186, 187), and targeting other immune checkpoint proteins and kinase signaling pathways in Tregs similarly disrupts their suppressive capacity (188). However, most investigations remain confined to preclinical studies or solid tumor trials, with AML-specific research notably limited. To realize effective Treg-targeted therapies in AML and maximize clinical benefits, comprehensive mechanistic elucidation and dedicated clinical validation are urgently required.

### 7.2 MDSCs in AML: current understanding and future directions

Similarly, insights into MDSC biology in AML may benefit greatly from an understanding of its mode of function in solid tumors and pan-cancer models. In TME, MDSCs highly express arginase-1 (ARG-1) (189) and inducible nitric oxide synthase (iNOS) (190), and transfer the metabolite methylglyoxal to CD8+ T cells (191), all of which degrade L-arginine and thus prevent T cell proliferation (192). In addition, MDSCs suppress T-cell activation by depleting cystine and cysteine (193). Within the TME, M-MDSCs exhibit heightened glucose uptake and consumption, thereby disrupting the metabolic activity of neighboring immune cells (194). Notably, in breast cancer models, MDSC-mediated tryptophan catabolism via IDO has been shown to drive Treg expansion while concurrently inducing T-cell autophagy, cell cycle arrest, and cell death (195). Adenosine production by CD39/CD73-expressing MDSCs further potentiates their expansion and enhances immunosuppressive activity in lung cancer models (196, 197). The immunosuppressive capacity of MDSCs is mediated through excessive generation of reactive oxygen species (ROS) (198, 199), nitric oxide (NO), and peroxynitrite (PNT) (200), which collectively impair T-cell function. Additionally, tumor-infiltrating MDSCs engage with T cells through multiple immune checkpoint interactions—including PD-L1/PD-1, Gal-9/TIM-3, CD80\CD86/CTLA-4, CD155/TIGIT, VISTA/VISTAL, and FasL/Fas—inducing T-cell anergy and apoptosis (201). In murine tumor models, tumor-expanded MDSCs can suppress NK cell function via membrane-bound TGF-β1 (202). However, the existence and relative contribution of these MDSC-mediated immunosuppressive mechanisms in AML remain unclear and warrant further investigation.

Therapeutic targeting of MDSCs represents a promising strategy to augment anti-leukemic immunity through multiple approaches: inhibiting their generation, promoting differentiation into immunocompetent mature cells, suppressing their immunosuppressive activity, or selectively depleting MDSC populations (203, 204). AML-derived EVs, characterized by surface palmitoylated proteins or c-Myc cargo, potently drive MDSC expansion (135, 142). EV inhibition represents a theoretically viable approach to curtail MDSC generation, and experimental validation remains essential. In addition, reprogramming existing MDSCs into immunocompetent mature cells serves as an alternative strategy. Preclinical studies demonstrate that all-trans retinoic acid (ATRA) effectively reprogram MDSCs into mature APCs, thereby restoring T-cell functionality in both renal carcinoma and pulmonary malignancy models (203, 205). Thus, pharmacological induction of MDSC differentiation into non-immunosuppressive myeloid lineages represents a viable therapeutic strategy for AML. The suppression of MDSC activity may be based on its immunosuppressive mechanisms, such as the reduction of ROS and NO production. Targeting depletion of MDSCs through agents like gemtuzumab ozogamicin (GO) has demonstrated significant clinical potential. As a CD33-directed antibody-drug conjugate (ADC) approved for CD33+ AML treatment, GO has shown both efficacy and a manageable safety profile in multiple clinical trials (206). The constitutive expression of CD33 across MDSC subtypes makes it an attractive therapeutic target, with a study by Fultang et al. demonstrating GO’s ability to increase MDSC death, consequently restoring T-cell response and enhancing tumor cell clearance (147). This study encompassed multiple tumor subtypes; however, AML samples were not included, warranting further investigation in the AML context. These findings provide a strong rationale for developing novel MDSC-targeted therapies in AML, potentially leading to significant advances in treatment outcomes.

### 7.3 LAMs in AML: current understanding and future directions

The AML microenvironment is characterized by significant infiltration of M2-like LAMs, which actively support leukemic cell survival and disease progression. These cells represent the leukemic counterpart of TAMs observed in solid malignancies. TAMs exhibit pro-tumorigenic properties through multiple mechanisms: (1) direct promotion of malignant cell proliferation and metastasis, (2) suppression of T cell-mediated anti-tumor immunity, and (3) facilitation of angiogenic processes. TAMs can facilitate the proliferation of tumor cells by producing growth factors, cytokines, and chemokines, including FGF-2, TGF-β, PDGF, IL-10, CXCL, and so on (207). Evidence demonstrates that TAMs significantly enhance osteosarcoma metastasis and invasion through activating the COX-2/STAT3 axis and epithelial-mesenchymal transition (208). TAMs suppress antitumor immunity by inhibiting T cells, B cells, NK cells, and DCs, while promoting Tregs, Th17, γδT cells, MDSCs, angiogenesis, and metastasis (207). TAMs can induce tumor angiogenesis through the secretion of cytokines, including VEGF, COX-2, and PDGF (209). Building on the well-characterized role of TAMs in solid tumors, investigating LAMs in AML represents a promising research direction.

Given the established pro-tumor functions of TAMs, targeting LAMs may offer novel therapeutic strategies to disrupt AML progression and improve treatment outcomes. Therapeutic reprogramming of LAMs from a pro-tumorigenic to an anti-tumor M1-like phenotype emerges as a promising strategy for AML treatment. Experimental evidence demonstrates that let-7b knockdown in LAMs induces M1-like polarization, resulting in significant suppression of AML progression and extended survival in MLL-AF9-driven murine leukemia models (156). RNA-seq profiling of AML patient-derived LAMs identified let-7b as a potential target, though its downstream mechanisms remain undefined. Future work should characterize let-7b effector pathways and assess whether targeting either the microRNA itself or its products offer therapeutic benefit in AML.

### 7.4 Bregs in AML: current understanding and future directions

Breg cells have been found to be increased in AML and are thought to be involved in the negative immunoregulation of the hematopoietic microenvironment of AML. However, so far, no specific marker has been identified for Breg cells to define their phenotype. These findings suggest that Breg cells may not represent a distinct lineage, but rather reflect a functional state adopted by B cells at various developmental stages in response to microenvironmental stimuli (169). Nevertheless, the possibility remains that specific Breg markers exist but were not identified in the current study. Further investigation is required to fully elucidate the origin, developmental pathways, and phenotypic characteristics of Breg cells. While their phenotype remains incompletely defined, their functional significance in immune regulation has become increasingly evident. Breg research in solid malignancies has revealed their critical immunosuppressive role, with IL-10 emerging as the prototypical functional marker of Breg (168, 210). Recent advances have revealed that Breg cells employ a broader immunomodulatory factor to mediate immune suppression, including TGF-β, IL-35, CD1d and PD-L1 (211). Breg cells suppress immune responses by inhibiting CD4+ T cell proliferation and cytokine secretion (212), while also blocking TNF-α production in monocyte-macrophages (172). Given the nascent state of Breg research in AML, systematic efforts are needed to map their ontogeny, functional heterogeneity, and clinical relevance. Such studies could unlock Breg-targeted therapies to complement existing AML immunotherapies.

### 7.5 LANs in AML: current understanding and future directions

While research on LANs in AML remains limited, their mechanistic roles in CLL have been well characterized (213). In CLL, LANs promote leukemic cell proliferation and survival via IL-17/IL-6 secretion while fostering immunosuppression through T-cell inhibition. Notably, LANs enhance bone marrow homing and maintain leukemic stemness via the CXCR4/CXCL12 axis (213, 214). These findings offer valuable insights for AML research, particularly regarding LANs-leukemic stem cell crosstalk and the therapeutic potential of modulating LANs polarization. Key unresolved questions include (1): spatiotemporal dynamics of LANs subsets in AML progression, and (2) mechanistic interactions between LANs and the leukemic stem cell niche. Addressing these gaps could advance precision immunotherapy strategies for AML.

### 7.6 The likely coordinated network of immunosuppressive cells in AML

**Figure 5:**
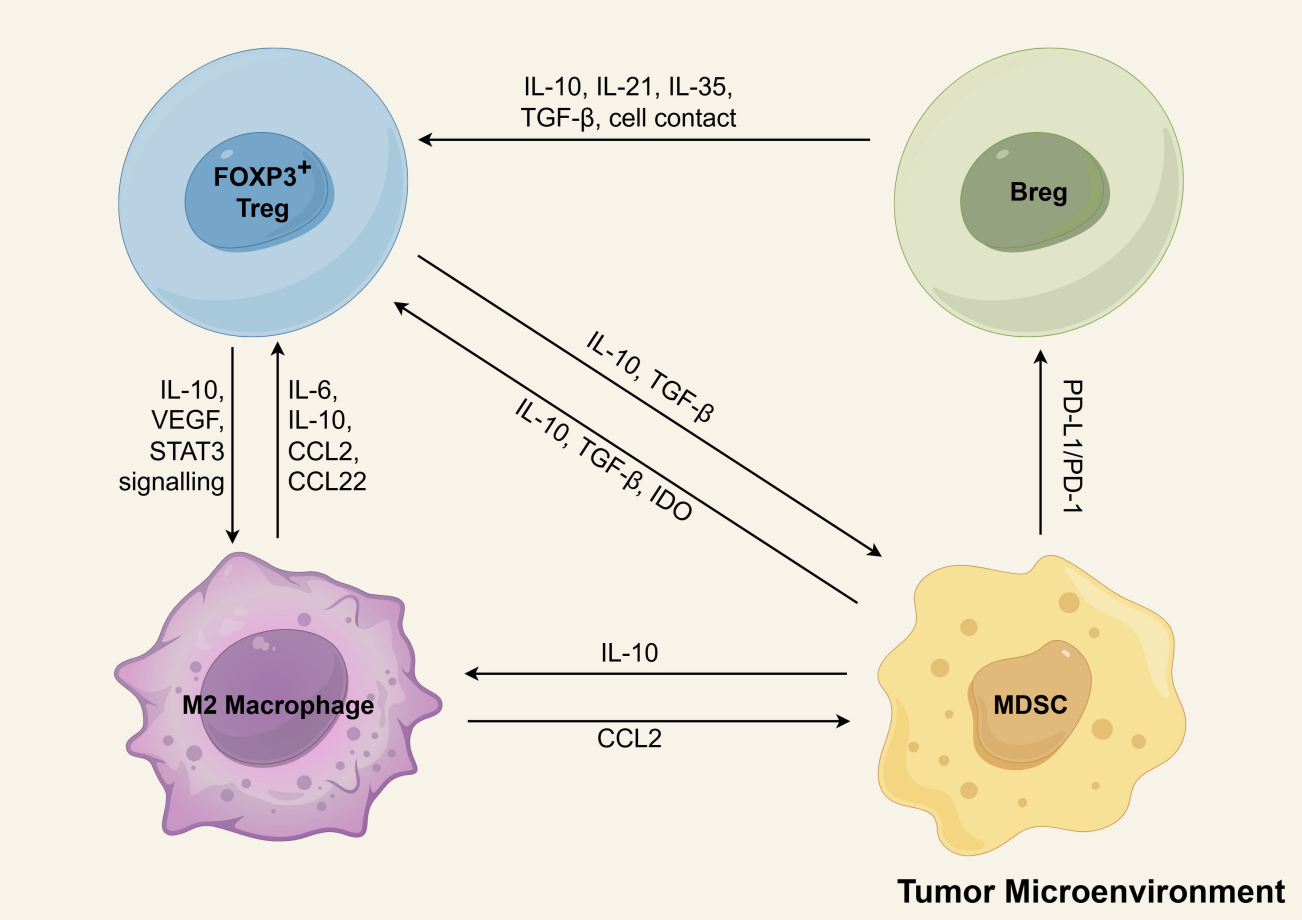
The positive feedback loops of immunosuppressive cells in tumor microenvironment. Tregs, MDSCs, M2 macrophages, and Bregs form interlinked positive feedback loops that reinforce immune suppression and drive immune evasion and AML progression. Key interactions include: (1) Breg-mediated enhancement of Treg function via IL-10, IL-21, IL-35, TGF-β, and direct cell contact; (2) Treg-induced monocyte-to-M2 differentiation through IL-10, VEGF, and STAT3 signaling; (3) M2 macrophage secretion of IL-6/IL-10 for Treg activation and CCL2/CCL22 for Treg recruitment; (4) Reciprocal TGF-β/IL-10-mediated activation between Tregs and MDSCs; (5) MDSC-driven Treg differentiation via IDO upregulation; (6) PD-1/PD-L1-dependent MDSC induction of PD-L1+ Bregs; and (7) IL-10-mediated MDSC promotion of M2 polarization. M2-derived CCL2 further recruits MDSCs to the TME. Schematic figure was drawn by Figdraw (www.figdraw.com).

The development of an immunosuppressive microenvironment in AML involves a coordinated interplay of multiple regulatory cell populations. While studies have individually characterized the leukemia-promoting effects of Tregs, MDSCs, LAMs, and Bregs, accumulating evidence suggests these cells function synergistically to establish a potent immunosuppressive network that facilitates immune evasion and disease progression (**Figure 5**). As demonstrated by Flores-Borja et al., CD19+CD24^high^CD38^high^ Bregs in healthy individuals can induce regulatory properties in CD4+CD25− T cells through IL-10-dependent mechanisms (212). However, in the study by Wan et al., the researchers observed that Bregs from healthy controls failed to promote the conversion of CD4+CD25− T cells into CD4+CD25+FOXP3+ Tregs, irrespective of whether the T cells originated from healthy individuals or AML patients. In contrast, BM-derived CD19+CD24^high^CD38^high^ Bregs of AML patients possessed this conversion capability. Furthermore, this conversion appeared to be primarily mediated through direct cell-to-cell contact, as cytokine profiling revealed no significant alterations in the expression levels of soluble factors (43). More investigations are required to elucidate the precise mechanisms underlying Treg and Breg interactions within the AML microenvironment. In the TME, it has been demonstrated that Bregs promote Treg tumorigenicity through secretion of IL-21, IL-35, and TGF-β (215). Emerging evidence demonstrates functional reciprocity between Tregs and MDSCs across diverse tumor models. This bidirectional crosstalk establishes self-reinforcing immunosuppressive circuits, wherein factors (such as TGF-β, IL-10) produced by each population reciprocally stimulate expansion and activation, thereby amplifying immune suppression within the TME (216). M-MDSCs in CLL exhibit elevated IDO expression, which drives enhanced Treg differentiation (217). In breast cancer, MDSCs promote the development of PD-L1+ Bregs through PD-1/PD-L1-mediated activation of the PI3K/AKT/NF-κB signaling axis in B lymphocytes (218). MDSCs can drive macrophage polarization toward an immunosuppressive M2-like phenotype via IL-10 secretion, thereby facilitating solid tumor progression (219). Additionally, M2 cells secrete CCL2 into the TME to recruit MDSCs and Tregs (220). A reciprocal regulatory axis further connects M2-polarized macrophages and Tregs within the TME (215). Tregs promote monocyte differentiation into M2 macrophages through the release of IL-10, VEGF and STAT3 signaling (215, 221). In turn, M2 cells secrete IL-6 (222) and IL-10 (223) to activate Tregs. M2 cells release CCL22 and recruit more CCR4-expressing Tregs to infiltrate the tumor microenvironment (224). Evidences suggests a coordinated network of immunosuppressive cells collectively fosters tumor progression in AML and other malignancies. While these cooperative mechanisms remain incompletely characterized, their systematic investigation represents a crucial frontier in tumor. A comprehensive elucidation of these cellular interactions potentially informing novel immunomodulatory approaches for AML.

### 7.7 Advantages and challenges of targeting immunosuppressive cells

#### 7.7.1 Advantages of targeting Tregs in AML immunotherapy

Targeting Tregs in AML immunotherapy offers multiple benefits. Depleting Tregs via anti-CD25 antibodies, IL-2DT, or CXCR4 inhibitors significantly enhances NK/CTL-mediated antileukemic activity, with preclinical studies demonstrating durable immune responses (124–126). Combination therapies (e.g., Treg depletion with DC vaccines) synergistically improve leukemic cell clearance (123), while clinical trials show that Treg-depleted donor lymphocyte infusions boost graft-versus-leukemia effects post-allo-HSCT (127, 128). Multiple targetable markers (LAG3/TIM3/CCR family) enable precise interventions, and existing regimens (e.g., IL-2DT) exhibit acceptable safety profiles (116).

#### 7.7.2 Challenges of targeting Tregs in AML immunotherapy

This approach faces critical limitations. Systemic Treg depletion risks triggering GVHD or autoimmune toxicity, and non-specific agents like CXCR4 inhibitors may compromise effector T cells. Tumor microenvironment complexity leads to compensatory immunosuppression (e.g., MDSC expansion) and drug delivery barriers, while Treg populations often rebound post-treatment. Clinical translation remains challenging, with current efficacy largely confined to murine models or post-transplant settings, limited responses in advanced AML, and a lack of predictive biomarkers for personalized therapy. These hurdles underscore the need for more precise Treg-targeting strategies and optimized combination regimens.

#### 7.7.3 Advantages of targeting MDSCs in AML immunotherapy

Targeting MDSCs in AML presents multiple therapeutic benefits, including the ability to reverse immunosuppression and restore anti-leukemic immune responses through various approaches such as CXCR4 inhibition (122), CD33-targeting agents (e.g., BiTE^®^ antibodies AMG 330 and TriKE^®^ engagers GTB-3550) (148, 149), and hypomethylating agents (150). These strategies have demonstrated efficacy in reducing MDSC populations and enhancing T-cell function in both preclinical models and early clinical trials. Additionally, combination therapies integrating MDSC-targeted interventions with chemotherapy or immune checkpoint blockade show synergistic effects, improving leukemic cell clearance and potentially overcoming treatment resistance (122).

#### 7.7.4 Challenges of targeting MDSCs in AML immunotherapy

However, MDSC-targeted therapies face significant hurdles, including the heterogeneity of MDSC subsets (e.g., M-MDSCs vs. PMN-MDSCs) with distinct immunosuppressive mechanisms, complicating broad-spectrum targeting. CD33-directed therapies may also deplete normal myeloid cells, leading to myelosuppression and infection risks. Furthermore, while preclinical studies are promising, clinical translation remains inconsistent, with variable patient responses and a lack of standardized biomarkers for patient selection. The tumor microenvironment’s adaptability, including compensatory recruitment of alternative immunosuppressive cells, further limits sustained efficacy, underscoring the need for more precise and combination-based strategies.

#### 7.7.5 Advantages of targeting LAMs in AML immunotherapy

Targeting LAMs in AML offers several therapeutic advantages. First, strategies such as CD169+/SIGLEC1+ macrophage depletion (165) and CD123/NKG2DL-targeted CAR-T therapy (149) have demonstrated significant efficacy in disrupting the immunosuppressive tumor microenvironment and directly eliminating pro-leukemic M2-like LAMs, leading to improved survival in preclinical models. Second, innovative approaches like TIGIT blockade (157) and let-7b knockdown (156) not only reduce M2 polarization but also actively reprogram LAMs toward anti-tumor M1 phenotypes, enhancing phagocytic activity and synergizing with therapies like anti-CD47. These dual-action mechanisms provide a multifaceted attack against AML progression while potentially restoring immune surveillance.

#### 7.7.6 Challenges of targeting LAMs in AML immunotherapy

Despite these advantages, LAM-targeted therapies face notable limitations. A major concern is the risk of off-target effects, as broad macrophage depletion may damage beneficial tissue-resident macrophages, potentially leading to unintended toxicity. Additionally, the plasticity of LAM phenotypes poses a challenge, as reprogrammed M1-like macrophages can revert to immunosuppressive M2 states under persistent tumor microenvironment pressures, undermining long-term therapeutic efficacy. Finally, while preclinical models (e.g., MLL-AF9-driven AML) show promise, translating these findings to human patients remains difficult due to the heterogeneity of LAM populations in AML and the lack of validated biomarkers for patient stratification. These hurdles highlight the need for more selective targeting strategies and robust combination approaches to maximize clinical benefit.

## 8 Conclusion

The therapeutic landscape of AML has been reshaped by immunotherapy advances, yet clinical outcomes remain suboptimal for most patients, with limited agents specifically targeting immunosuppressive cells. Critical challenges endure in characterizing these inhibitory immune populations, as key molecular signatures for distinct subsets remain undefined. While preclinical studies constitute most current research, few therapeutic strategies have advanced to clinical testing, highlighting crucial unmet needs in bridging the laboratory-to-clinic translation gap for immunotherapeutic development.
