# Supplementary material for: Immunosuppressive cells in acute myeloid leukemia: mechanisms and therapeutic target
Source: Front Immunol. 2025 Jul 23;16:1627161. doi: 10.3389/fimmu.2025.1627161 (PMC12325080; doi:10.3389/fimmu.2025.1627161)
Supplement: Supplementary file 1 [file Table1.docx]

# Supplementary Material for Tables for Immunosuppressive Cells in Acute Myeloid Leukemia: Mechanisms and Therapeutic Target

Mengnan Liu^1†^, Mengting Yang^2,3†^, Yue Qi^2,3†^, Yuting Ma^2,3†^, Qulian Guo^2,3^, Ling Guo^2,3^, Chunyan Liu^2,3^, Wenjun Liu^2,3*^, Lan Xiao ^2,3*^, You Yang^2,3*^

^1^ Department of Cardiovascular Medicine, Affiliated Traditional Chinese Medicine Hospital, Southwest Medical University, Luzhou 646000, China.

^2^ Department of Pediatrics (Children Hematological Oncology), Birth Defects and Childhood Hematological Oncology Laboratory, The Affiliated Hospital of Southwest Medical University, Sichuan Clinical Research Center for Birth Defects, Luzhou, Sichuan, 646000, China.

^3^ Department of Pediatrics, Southwest Medical University, Luzhou, Sichuan, China.

† These authors contributed equally to this work.

*** Corresponding Author:**

Wenjun Liu,

Department of Pediatrics (Children Hematological Oncology), Birth Defects and Childhood Hematological Oncology Laboratory, The Affiliated Hospital of Southwest Medical University, Sichuan Clinical Research Center for Birth Defects, Luzhou, Sichuan, 646000, China;

Phone number: +86 830 3165943;

E-mail: [wenjun_liu@swmu.edu.cn](mailto:wenjun_liu@swmu.edu.cn)

Lan Xiao,

Department of Pediatrics (Children Hematological Oncology), Birth Defects and Childhood Hematological Oncology Laboratory, The Affiliated Hospital of Southwest Medical University, Sichuan Clinical Research Center for Birth Defects, Luzhou, Sichuan, 646000, China;

Phone number: +86 830 3165943;

E-mail: 814510851@qq.com

You Yang,

Department of Pediatrics (Children Hematological Oncology), Birth Defects and Childhood Hematological Oncology Laboratory, The Affiliated Hospital of Southwest Medical University, Sichuan Clinical Research Center for Birth Defects, Luzhou, Sichuan, 646000, China;

Phone number: +86 830 3165943;

E-mail: [youyang091@swmu.edu.cn](mailto:youyang091@swmu.edu.cn);

#

**Supplementary Table S1. The studies on Treg accumulation in AML patients.**

| **Author, year** | **Number of patients** | **Time of sampling** | **Source of sampling** | **Phenotype definition** | **Conclusions** | **Reference** |
| --- | --- | --- | --- | --- | --- | --- |
| Wang, 2005 | 36 | ND | PB & BM | CD4+CD25^high^ | The frequency of CD4+CD25^high^Treg in PB of AML patients is significantly higher when compared with healthy individuals. | (1) |
| Szczepanski, 2009 | 31 | ND | PB | CD4+CD25^high^ | Treg frequency correlated with response to induction chemotherapy.  Treg are resistant to conventional chemotherapy. | (2) |
| Ersvaer, 2010 | 20 | ND & during chemotherapy | PB | CD4+CD25+FOXP3+ | Relative level of circulating CD4+CD25+FOXP3+ T cell is increased in AML patients before and following intensive chemotherapy. | (3) |
| Zhang, 2010 | 182 | ND & CR | PB & BM | CD4+CD25+CD127^low^ & CD4+CD25^high^ | Elevated frequencies of CD4+CD25+CD127^low^ and CD4+CD25^high^Treg in patients with AML.  Treg frequencies decreased in PB and BM of patients when acquired CR. | (4) |
| Kanakry, 2011 | 20 | During chemotherapy | PB | CD4+CD25+FOXP3+ | Recovering T lymphocytes included a greatly expanded population of CD4+CD25+FOXP3+ T cells. | (5) |
| Yang, 2013 | 80 | ND & CR & relapse | PB | CD4+CD25+CD127^low^ | Treg number in PB could be used to monitor disease status and evaluate disease progression. | (6) |
| Lichtenegger, 2014 | 17 | During chemotherapy | PB | CD4+CD25+FOXP3+CD127− | During the course of maintenance therapy, Treg counts increase. | (7) |
| Tian, 2015 | 48(34, 19) ^a^ | ND & CR & relapse | BM | CD4+CD25+FOXP3+ | Treg cells elevated and imbalanced Th17/Treg in BM of AML patients. | (8) |
| Sander, 2017 | 84 | CR | PB | CD4+CD14−CD25^high^CD127^low^ | Immunotherapy with HDC/IL-2 in AML entails induction of immunosuppressive Tregs that may be targeted for improved anti-leukemic efficiency. | (9) |
| Guo, 2020 | 22 | ND | PB | CD4+CXCR5+PD-1+FOXP3+ | Higher Percentage of cTfr cells in AML Patients.  Significantly lower percentages of cTfr cells were found in the group of AML patients with CR. | (10) |
| Jin, 2020 | 27(7, 12) ^b^ | ND & non-remission & CR | PB | FOXP3+γδTCR+ | γδ Treg cells are significantly higher in de novo and NR patients. | (11) |
| Wan, 2020 | 45 | ND | PB & BM | CD4+CD25+CD127^low/−^ | Increased frequencies of Tregs in AML patients.  Enhanced migratory capacity of Tregs due to increased expression of CXCR4. | (12) |
| Zheng, 2022 | 36 | ND | PB | PD-1+FOXP3+γδTCR+ | A significant increase in the PD-1+FOXP3+γδT cell subset in AML was associated with poor clinical outcome. | (13) |
| Zhang, 2024 | 30(25)^c^ | ND & CR | BM | CD4+CD25+CD127^low/−^ | PD-1+ Tregs accumulation in BM in higher leukemic burden setting was linked to lactate acid secreted by AML blasts and decreased after disease remission. | (14) |

a: 48 newly-diagnosed, 34 complete-remission achieved and 19 relapsed-refractory patients

b: 27 newly-diagnosed, 7 non-remission and 12 complete-remission achieved patients

c: 30 newly-diagnosed, 25 complete-remission achieved patients

ND: newly diagnosed, CR: complete remission, PB: periphery blood, BM: bone marrow

# References

1. Wang X, Zheng J, Liu J, Yao J, He Y, Li X, et al. Increased Population of Cd4(+)Cd25(High), Regulatory T Cells with Their Higher Apoptotic and Proliferating Status in Peripheral Blood of Acute Myeloid Leukemia Patients. *Eur J Haematol* (2005) 75(6):468-76. doi: 10.1111/j.1600-0609.2005.00537.x.

2. Szczepanski MJ, Szajnik M, Czystowska M, Mandapathil M, Strauss L, Welsh A, et al. Increased Frequency and Suppression by Regulatory T Cells in Patients with Acute Myelogenous Leukemia. *Clin Cancer Res* (2009) 15(10):3325-32. Epub 20090505. doi: 10.1158/1078-0432.Ccr-08-3010.

3. Ersvaer E, Liseth K, Skavland J, Gjertsen BT, Bruserud Ø. Intensive Chemotherapy for Acute Myeloid Leukemia Differentially Affects Circulating Tc1, Th1, Th17 and Treg Cells. *BMC Immunol* (2010) 11:38. Epub 20100709. doi: 10.1186/1471-2172-11-38.

4. Shenghui Z, Yixiang H, Jianbo W, Kang Y, Laixi B, Yan Z, et al. Elevated Frequencies of Cd4⁺ Cd25⁺ Cd127lo Regulatory T Cells Is Associated to Poor Prognosis in Patients with Acute Myeloid Leukemia. *Int J Cancer* (2011) 129(6):1373-81. Epub 20110226. doi: 10.1002/ijc.25791.

5. Kanakry CG, Hess AD, Gocke CD, Thoburn C, Kos F, Meyer C, et al. Early Lymphocyte Recovery after Intensive Timed Sequential Chemotherapy for Acute Myelogenous Leukemia: Peripheral Oligoclonal Expansion of Regulatory T Cells. *Blood* (2011) 117(2):608-17. Epub 20101008. doi: 10.1182/blood-2010-04-277939.

6. Yang W, Xu Y. Clinical Significance of Treg Cell Frequency in Acute Myeloid Leukemia. *Int J Hematol* (2013) 98(5):558-62. Epub 20131019. doi: 10.1007/s12185-013-1436-3.

7. Lichtenegger FS, Lorenz R, Gellhaus K, Hiddemann W, Beck B, Subklewe M. Impaired Nk Cells and Increased T Regulatory Cell Numbers During Cytotoxic Maintenance Therapy in Aml. *Leuk Res* (2014) 38(8):964-9. Epub 20140602. doi: 10.1016/j.leukres.2014.05.014.

8. Tian T, Yu S, Liu L, Xue F, Yuan C, Wang M, et al. The Profile of T Helper Subsets in Bone Marrow Microenvironment Is Distinct for Different Stages of Acute Myeloid Leukemia Patients and Chemotherapy Partly Ameliorates These Variations. *PLoS One* (2015) 10(7):e0131761. Epub 20150702. doi: 10.1371/journal.pone.0131761.

9. Sander FE, Nilsson M, Rydström A, Aurelius J, Riise RE, Movitz C, et al. Role of Regulatory T Cells in Acute Myeloid Leukemia Patients Undergoing Relapse-Preventive Immunotherapy. *Cancer Immunol Immunother* (2017) 66(11):1473-84. Epub 20170718. doi: 10.1007/s00262-017-2040-9.

10. Guo Z, Chen Z, Xu Y, Zhang Y, Hu L, Yu F, et al. The Association of Circulating T Follicular Helper Cells and Regulatory Cells with Acute Myeloid Leukemia Patients. *Acta Haematol* (2020) 143(1):19-25. Epub 20190618. doi: 10.1159/000500588.

11. Jin Z, Ye W, Lan T, Zhao Y, Liu X, Chen J, et al. Characteristic of Tigit and Dnam-1 Expression on Foxp3+ Γδ T Cells in Aml Patients. *Biomed Res Int* (2020) 2020:4612952. Epub 20200727. doi: 10.1155/2020/4612952.

12. Wan Y, Zhang C, Xu Y, Wang M, Rao Q, Xing H, et al. Hyperfunction of Cd4 Cd25 Regulatory T Cells in De Novo Acute Myeloid Leukemia. *BMC Cancer* (2020) 20(1):472. Epub 20200526. doi: 10.1186/s12885-020-06961-8.

13. Zheng J, Qiu D, Jiang X, Zhao Y, Zhao H, Wu X, et al. Increased Pd-1(+)Foxp3(+) Γδ T Cells Associate with Poor Overall Survival for Patients with Acute Myeloid Leukemia. *Front Oncol* (2022) 12:1007565. Epub 20221215. doi: 10.3389/fonc.2022.1007565.

14. Zhang Y, Huang Y, Hong Y, Lin Z, Zha J, Zhu Y, et al. Lactate Acid Promotes Pd-1(+) Tregs Accumulation in the Bone Marrow with High Tumor Burden of Acute Myeloid Leukemia. *Int Immunopharmacol* (2024) 130:111765. Epub 20240305. doi: 10.1016/j.intimp.2024.111765.
